# Elucidating the putative link between prefrontal neurotransmission, functional connectivity, and affective symptoms in irritable bowel syndrome

**DOI:** 10.1038/s41598-019-50024-3

**Published:** 2019-09-19

**Authors:** Adriane Icenhour, Sofie Tapper, Olga Bednarska, Suzanne T. Witt, Anders Tisell, Peter Lundberg, Sigrid Elsenbruch, Susanna Walter

**Affiliations:** 1 Department of Gastroenterology and Department of Clinical and Experimental Medicine, Linköping University, Linköping, Sweden; 2 Center for Medical Image Science and Visualization (CMIV), Linköping University, Linköping, Sweden; 3 Department of Medical and Health Sciences, Linköping University, Linköping, Sweden; 4 Department of Radiation Physics, Department of Medical and Health Sciences, Linköping University, Linköping, Sweden; 5 Department of Radiology, Department of Medical and Health Sciences, Linköping University, Linköping, Sweden; 6 Institute of Medical Psychology and Behavioral Immunobiology, University Hospital Essen, University of Duisburg-Essen, Essen, Germany

**Keywords:** Human behaviour, Comorbidities

## Abstract

Altered neural mechanisms are well-acknowledged in irritable bowel syndrome (IBS), a disorder of brain-gut-communication highly comorbid with anxiety and depression. As a key hub in corticolimbic inhibition, medial prefrontal cortex (mPFC) may be involved in disturbed emotion regulation in IBS. However, aberrant mPFC excitatory and inhibitory neurotransmission potentially contributing to psychological symptoms in IBS remains unknown. Using quantitative magnetic resonance spectroscopy (qMRS), we compared mPFC glutamate + glutamine (Glx) and γ-aminobutyric acid (GABA+) concentrations in 64 women with IBS and 32 age-matched healthy women (HCs) and investigated their association with anxiety and depression in correlational and subgroup analyses. Applying functional magnetic resonance imaging (fMRI), we explored whether altered neurotransmission was paralleled by aberrant mPFC resting-state functional connectivity (FC). IBS patients did not differ from HCs with respect to mPFC GABA+ or Glx levels. Anxiety was positively associated with mPFC GABA+ concentrations in IBS, whereas Glx was unrelated to psychological or gastrointestinal symptoms. Subgroup comparisons of patients with high or low anxiety symptom severity and HCs revealed increased GABA+ in patients with high symptom severity, and lower mPFC FC with adjacent anterior cingulate cortex (ACC), a crucial region of emotion modulation. Our findings provide novel evidence that altered prefrontal inhibitory neurotransmission may be linked to anxiety in IBS.

## Introduction

The relevance of bidirectional communication pathways between the brain and the gastrointestinal system, the brain-gut-axis, is increasingly acknowledged in health and in the pathophysiology of various somatic and neuropsychiatric disturbances^1^. Irritable bowel syndrome (IBS) is considered an exemplary disorder of brain-gut communication. Cardinal symptoms of the heterogeneous disease are gastrointestinal in nature, yet psychiatric comorbidities particularly with anxiety and depression affect a large proportion of IBS patients^2,3^, with profound clinical implications^4–6^. While this key role of psychological symptoms in altered brain-gut communication is increasingly appreciated, distinct neural correlates of anxiety and depression symptoms in IBS are only beginning to be elucidated.

Functional magnetic resonance imaging (fMRI) studies frequently demonstrate aberrant brain function involving regions associated with emotion processing and regulation in IBS^7^. Within these circuits, the medial prefrontal cortex (mPFC) including rostral anterior cingulate cortex (rACC) is among the regions with most consistently observed functional alterations^7,8^. The mPFC represents a key node in emotion regulation and top-down corticolimbic inhibitory control^9^. In line with this, observed changes in mPFC function in IBS are reportedly associated with symptoms of anxiety and depression^10–12^. Dysfunctional prefrontal inhibitory control may therefore be a crucial mechanism underlying aberrant emotion regulation and psychological symptoms in IBS. Notwithstanding these well-documented functional mPFC alterations in IBS, their putative neurochemical basis remains largely unknown.

As crucial biochemical markers of brain tissue excitation and inhibition^13^, glutamate (Glu, often reported as a combined glutamate + glutamine measurement, Glx) and γ-aminobutyric acid (GABA, commonly reported with a co-edited macromolecular signal as GABA+^14^) may be key players in altered brain function in IBS. A previous quantitative magnetic resonance spectroscopy (qMRS) study described reductions of hippocampal Glu^15^ and recent findings document lower Glx concentrations in bilateral insulae in IBS patients^16^, extending evidence of aberrant neurotransmission assessed from different brain regions in pelvic pain^17^ and fibromyalgia^18^, diseases highly comorbid with IBS^2^. Reported associations between neurotransmitter concentrations and psychological symptoms^15,18,19^ lend first support for a relation between psychological factors and central alterations, even on a biochemical level. These data are substantiated by altered biochemistry within different target regions observed in patients with anxiety disorders and depression^20–22^. Finally, recent data document a link between anxiety, increased mPFC GABA+ concentrations and changes in functional connectivity (FC) in healthy volunteers^23,24^, supporting a distinct role of increased prefrontal GABA in disturbed corticolimbic inhibition^25^. A dysregulation of prefrontal inhibitory control, possibly due to an abundance of GABA inhibiting regulatory circuits, may thus be involved in aberrant emotion regulation and increased psychological complaints^25^, which remains to be elucidated in patients with IBS.

In this combined qMRS and resting-state fMRI study, we therefore aimed to investigate mPFC Glx and GABA+ concentrations and their relation to psychological as well as gastrointestinal symptoms in female patients with IBS and age-matched healthy women. We hypothesized IBS patients to exhibit dysregulated mPFC neurotransmitter levels, particularly increased GABA+. We further expected symptoms of anxiety and/or depression to be related to aberrant prefrontal neurotransmitter concentrations and tested in subgroup analyses, whether biochemical alterations were most pronounced in patients with high severity of anxiety/depression. Finally, we explored FC of mPFC with amygdala as a core node of corticolimbic circuitry^24,25^, and with insula and ACC as key players in emotion processing and modulation with a crucial role in both, anxiety^26^ and IBS^7,8^. We hypothesized disrupted connectivity within these circuits particularly in patients with a high severity of anxiety/depression.

## Results

### Sample characteristics

Patients presented with moderate to severe IBS and were characterized by greater symptom-specific anxiety, higher pain intensity, and more interference compared to HCs (all *p* < 0.001; Supplementary Table S1). Patients further exhibited higher severity of anxiety and depression symptoms (all *p* < 0.001).

### Between-group comparisons of mPFC neurotransmitter levels

**Figure 1:**
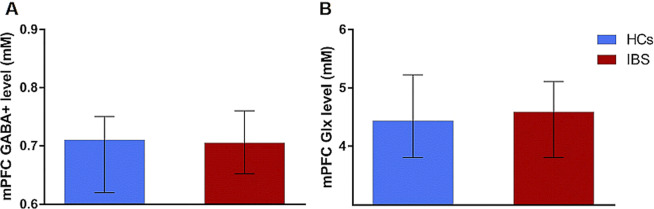
Group comparisons of mPFC GABA+ (**A**) and Glx (**B**) concentrations in HCs (N = 32; blue) and patients with IBS (N = 64; red). Data are shown as median and error bars indicate interquartile ranges.

Compared to HCs, IBS patients in general did not show significant differences in concentrations of either mPFC GABA+ (*U* = 933.50; *p* = 0.488) or Glx (*U* = 979.50; *p* = 0.728) (Fig. 1). Notably, a proportion of patients used selective serotonin-reuptake inhibitors or low-dose tricyclic antidepressants at the time of participation. To exclude potential confounding effects of centrally-acting medication, we conducted additional between-group analyses of qMRS data including only patients free from antidepressants (N = 43). Results confirmed no between-group differences in GABA+ or Glx concentrations (Supplementary Notes S2a). Exploratory analyses of mPFC total N-acetylaspartate (tNA), Creatine (tCr) and Choline (Cho) further revealed no differences between patients and controls (all *p* > 0.10; Supplementary Table S3).

### Associations between neurotransmitter concentrations and psychological symptoms

**Figure 2:**
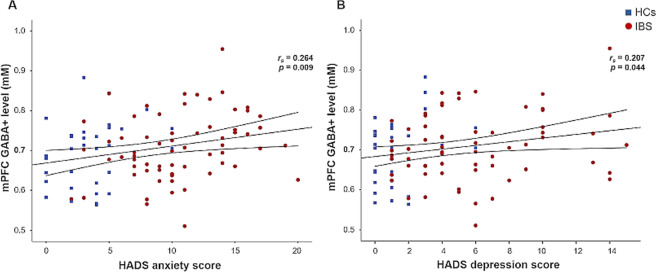
Spearman’s rank correlations of mPFC GABA+ concentrations with (**A**) HADS anxiety scores and (**B**) HADS depression scores in all subjects. Patients with IBS (N = 64) are depicted as blue squares, HCs (N = 32) as red circles.

Correlational analyses in the full sample (Supplementary Table S4) and in patients and HCs separately were conducted to investigate whether concentrations of mPFC neurotransmitters were associated with symptoms of anxiety and depression. We further explored associations with disease-related measures, such as symptom severity, pain intensity and interference, and GI symptom-specific anxiety to estimate the relative specificity of our findings to psychological symptoms. Briefly, in the full sample GABA+ concentrations were most strongly associated with anxiety symptoms (*r*_*s*_ = 0.264; *p* = 0.009; Fig. 2A) and showed weaker correlations with symptoms of depression (*r*_*s*_ = 0.207; *p* = 0.044, Fig. 2B), whereas Glx was not associated with psychological symptoms. Analyses conducted in IBS patients and HCs separately substantiated a distinct association between mPFC GABA+ concentrations and anxiety in patients (*r*_*s*_ = 0.293; *p* = 0.019), whereas the correlation with depression did not yield significance and psychological symptoms were not correlated with GABA+ in HCs. Prefrontal Glx levels showed no association with anxiety or depression in either group. Neurotransmitter concentrations in mPFC did not correlate with any disease-related measure. Exploratory correlational analyses regarding associations of anxiety and depression with tNA, tCr and Cho revealed no significant results (all *p* > 0.242; data not shown). Based on correlational findings suggesting distinct associations between mPFC GABA+ concentrations and anxiety in patients, GABA+ was solely considered for further analyses and we focused on anxiety symptoms in subsequent subgroup analyses with patient subgroups based on HADS anxiety scores, as described below.

#### Definition of IBS subgroups based on anxiety scores

**Figure 3:**
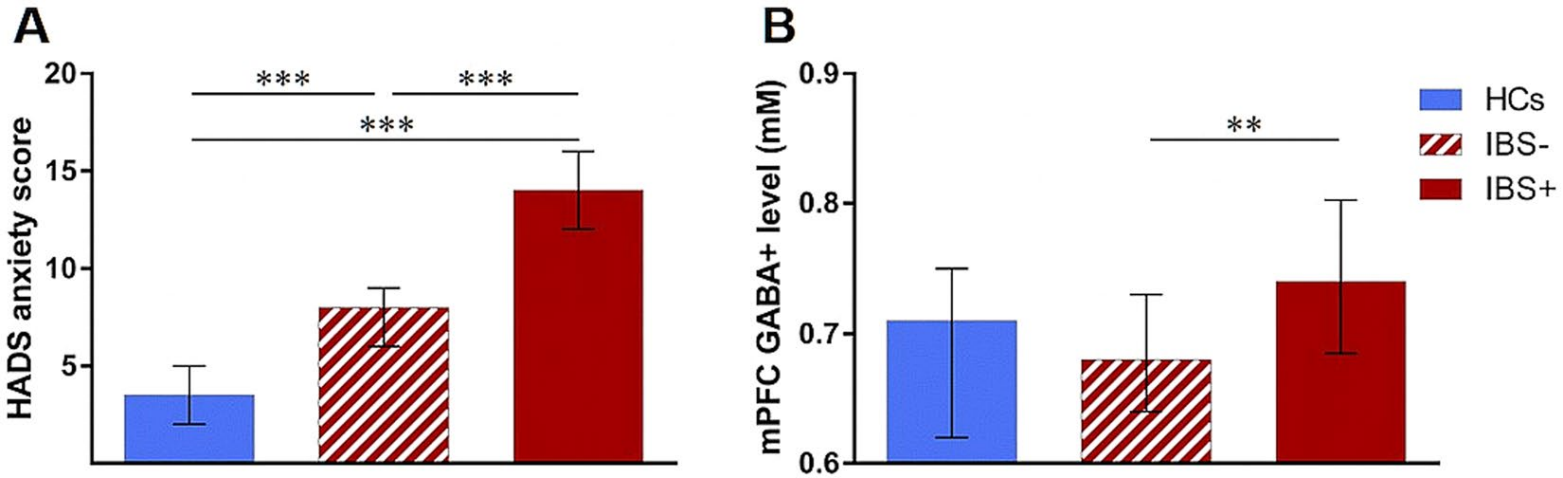
Group differences in HADS anxiety scores (**A**) and mPFC GABA+ concentrations (**B**) between HCs (N = 32; solid blue), IBS patients with low severity of anxiety symptoms (N = 34; red striped) and patients with high severity of anxiety symptoms (N = 30; solid red). Data are given as median and error bars indicate interquartile ranges. **p < 0.01; ***p < 0.001.

To further elucidate the relationship between anxiety symptoms and prefrontal GABA+ levels, the patient sample was subdivided according to low (IBS −; N = 34) or high (IBS +; N = 30) severity of anxiety symptoms. In addition to more symptoms of anxiety (IBS+: M = 14.00 (12.00–16.00); IBS−: M = 8.00 (6.00–9.25); Fig. 3A), the IBS+ subgroup revealed more depression symptoms relative to IBS− (IBS+: M = 7.50 (4.00–10.75); IBS−: M = 4.00 (2.00–6.00)) (both *p* < 0.001). Patients with high severity of anxiety symptoms reported higher pain intensity and interference (both *p* < 0.05), whereas IBS symptom severity and symptom-specific anxiety were comparable.

#### *Subgroup analyses of mPFC* GABA+ *concentrations*

Kruskal-Wallis test revealed a significant effect of group on mPFC GABA+ concentrations (*X*^2^_(2)_ = 8.346; *p* = 0.014). Post hoc *U*-tests demonstrated significantly higher GABA+ concentrations in the IBS+ relative to the IBS− subgroup (*U* = 299.50; *p* = 0.004) and to HCs (*U* = 336.50; *p* = 0.045; Fig. 3B), the latter failing statistical significance after Bonferroni correction. No differences in GABA+ concentrations were observed between IBS− and HCs. On removing data from patients taking antidepressant medication, subgroup analyses yielded similar results (Supplementary Notes S2b).

### Analyses of resting-state FC

**Figure 4:**
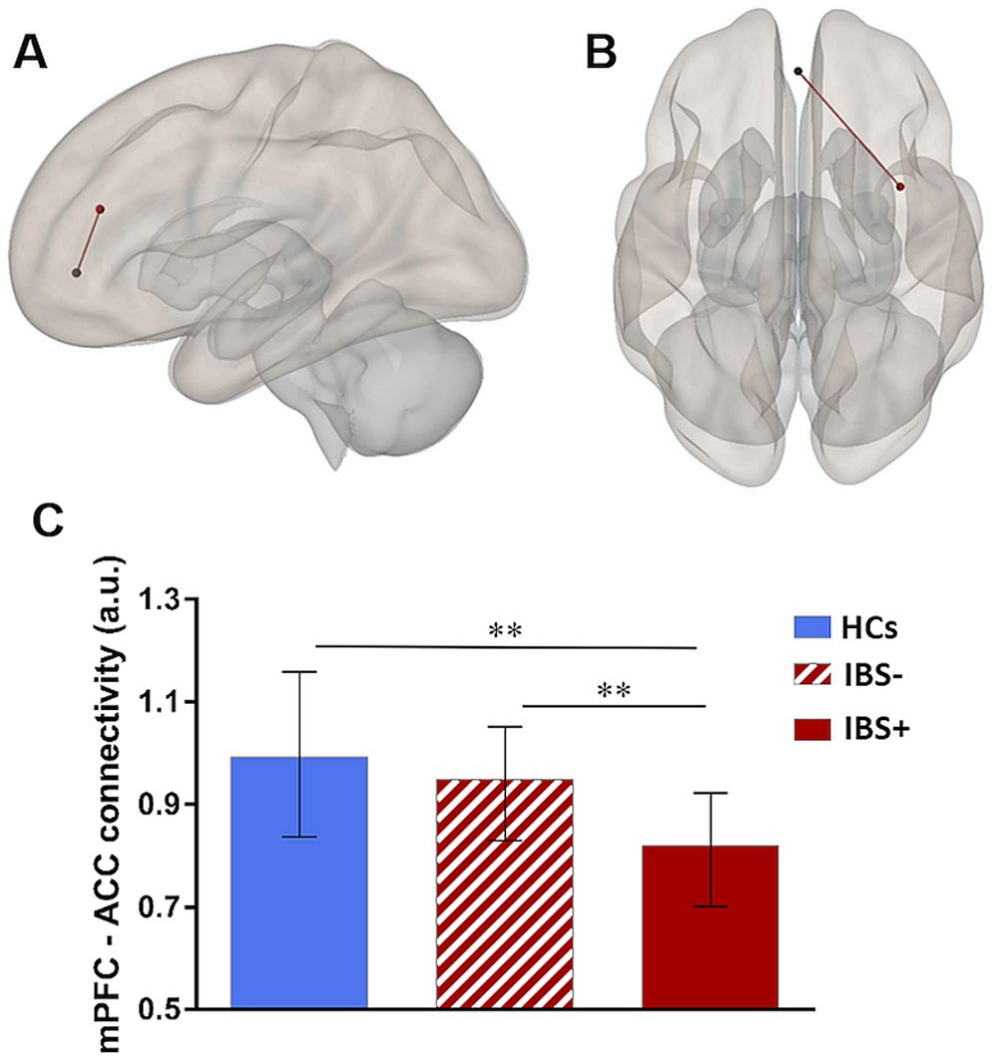
Results from group comparisons of resting-state FC between mPFC and ACC (**A**) and between mPFC and insula (**B**) (HCs > IBS). (**C**) Subgroup comparisons of FC in patients with high severity of anxiety symptoms (IBS+; N = 30; solid red), patients with low anxiety symptom severity (IBS−; N = 33; red striped) and HCs (N = 32; solid blue). Data are given as median and error bars indicate interquartile ranges. **p < 0.01.

To determine whether the association between biochemical alterations in mPFC and symptoms of anxiety was paralleled by altered FC, exploratory analyses of connectivity

from mPFC to amygdala, ACC and insula, respectively, were conducted. Patients showed significantly reduced FC between mPFC and ACC (t = 2.36; *p*_FDR_ = 0.02; Fig. 4A) and between mPFC and right insula relative to HCs (t = 2.37; *p*_FDR_ = 0.02; Fig. 4B), whereas no group differences were observed in mPFC – amygdala FC (data not shown). These group differences were confirmed by analyses on extracted mean FC values (ACC: *U* = 712.00; *p* = 0.018; right insula: *U* = 755.00; *p* = 0.047). Exploratory correlational analyses suggested connectivity between mPFC and ACC to be distinctly related to anxiety in IBS (*r*_*s*_ = −0.356; *p* = 0.004), while effects for FC between mPFC and insula or amygdala failed statistical significance. Based on between-group and correlational findings, subgroup analyses focused on mPFC – ACC FC. Kruskal-Wallis test comparing mean FC in IBS+ , IBS−, and HCs revealed a significant effect of group (*X*^2^_(2)_ = 13.71; *p* < 0.001). Post-hoc *U*-tests confirmed reduced mPFC – ACC connectivity in IBS patients with high when compared to the subgroup with low anxiety symptom severity (*U* = 273.00; *p* = 0.002) and to HCs (*U* = 234.00; *p* = 0.001), while IBS− did not differ from HCs (Fig. 4C).

### Association between mPFC GABA+, FC and anxiety in patients

Finally, to explore the predictive value of mPFC FC and GABA+ concentrations for anxiety severity in IBS patients, exploratory multiple regression analyses were conducted, entering mPFC – ACC FC and GABA+ as predictors. Analyses revealed both, higher GABA+ (ß = 0.272; t = 2.35; *p* = 0.022) and decreased mPFC - ACC FC (ß = −0.323; t = −2.78; *p* = 0.007) to be independent predictors of anxiety in patients with IBS. The significant model including both measures (F = 6.59; *p* = 0.003) accounted for almost 18% of variance in anxiety severity (R^2^ for separate regressors: GABA+ = 0.074; mPFC FC = 0.103).

## Discussion

This multimodal study provides novel evidence of increased GABAergic neurotransmitter levels in mPFC, a core node of inhibitory control with a crucial role in emotion regulation, in women with IBS suffering from high severity of anxiety symptoms. Higher anxiety in patients was further associated with reduced FC between mPFC and adjacent ACC and both, increased GABA+ and reduced connectivity independently predicted higher anxiety symptom severity. These findings suggest that both, a disruption of inhibitory neurotransmission and blunted FC within prefrontal regulatory circuits may be involved in increased severity of anxiety frequently observed in IBS. Importantly, alterations in GABA concentrations were only evident in patients with high, not those with lower severity of anxiety symptoms and were not associated with depression, pain or GI symptoms. Therefore, our data suggest an effect distinctly associated with anxiety rather than the chronic pain condition itself.

Disturbances in GABA-mediated inhibitory control, likely via dysfunctional top-down modulation, are increasingly acknowledged in the pathophysiology of anxiety disorders^25^. Reduced inhibitory modulation appears to be associated with hyper-excitability of limbic regions that mediate emotional responses, conceivably resulting in an exaggerated expression of fear and anxiety^25^. The close relationship between prefrontal GABA, functional coupling within corticolimbic circuits, and anxiety has recently been demonstrated in healthy volunteers^23,24^. It is further consistent with hypo-activity in prefrontal regions, paralleled by hyper-activation of subcortical areas associated with the experience and expression of anxiety in posttraumatic stress disorder^26^. Our findings substantially extend this evidence by implicating disturbances in inhibitory neurotransmission as a biochemical underpinning of disturbed emotion processing and regulation and a key mechanism potentially underlying increased comorbidity with anxiety also in IBS. They further provide novel insights into the close relation between frequently reported functional brain alterations and psychological symptoms in IBS patients^10–12^. Specifically, in patients with high anxiety symptom severity, we observed decreased mPFC connectivity with ACC, a core node of the limbic system with a unique integrative role in emotion regulation^27,28^. These observations complement previous reports of abnormal top-down modulation in IBS and other disorders of brain-gut-communication^29,30^. Of note, regression findings suggested these alterations on functional and biochemical levels in patients with IBS to be independent rather than directly linked phenomena. Together, our multimodal findings support a model of excessive GABA-mediated inhibition of mPFC and compromised prefrontal connectivity, which both might contribute to a failure of effectively engaging regulatory processes and to dysfunctional inhibitory control as mechanisms involved in increased anxiety symptom severity in IBS.

While higher mPFC GABA+ concentrations in IBS patients with increased severity of anxiety supported our hypothesis, we did not observe altered Glx levels in patients. Reduced hippocampal excitatory neurotransmitter concentrations have been reported in a single early qMRS study in IBS^15^. A recently published study reporting reduced Glx in bilateral insulae and a hemisphere-specific association with pain and pain coping^16^ extends the, to date, scarce evidence in this patient group. Furthermore, evidence of altered glutamatergic neurotransmission involving various brain regions in conditions highly comorbid with IBS such as pelvic pain^17^, fibromyalgia^18^, as well as anxiety disorders^21,31^ and depression^20^ is accumulating. Regarding GABA+, a recent investigation of neurotransmitter levels in women with chronic pelvic pain reported lowered concentrations of GABA+ in ACC of patients, a region adjacent to mPFC. The authors further presented associations of ACC choline, but not GABA+ concentrations, with connectivity to regions of the limbic system and with negative mood^32^. At the same time, work in healthy volunteers specifically targeting mPFC neurotransmission rather supported a distinct association between GABA+, not Glx, and anxiety^23^, consistent with our observations. As recently proposed, these partly inconsistent findings on neurotransmission in the human brain might be attributable to disease-, symptom- as well as region-specific alterations^33^. Supporting this assumption, we did not find associations between mPFC GABA+ and disease-related measures such as GI symptom severity or pain, whereas a previous observation from our laboratory suggested a link between insular Glx with pain and pain coping in IBS, yet no association with emotional factors and no differences in GABA+ between patients and controls^16^. Furthermore, while patients with high severity of anxiety symptoms also exhibited elevated severity of depression, two symptom clusters with large overlap^34^, anxiety but not depression symptoms appeared to be distinctly associated with GABA levels in patients. Our findings might therefore suggest altered prefrontal GABA to be a mechanism specific to anxiety rather than the chronic pain condition or psychological symptoms in general. Such specificity might further explain comparable neurotransmitter levels between female patients in general and healthy women, yet distinct alterations in subgroups based on anxiety symptom severity observed herein, further underscoring IBS heterogeneity^35^.

Finally, despite the observed association between altered inhibitory neurotransmission and anxiety, we did not find an association between symptoms of anxiety and mPFC – amygdala FC in IBS patients. While amygdala is considered a key region of the limbic system with crucial relevance in anxiety disorders^25^, recent evidence supports a broader network including ACC and insula along with prefrontal cortex in the pathophysiology of emotional disturbances^9,26^. Functional alterations in ACC and insula are among the most consistently reported brain imaging findings in IBS, whereas a specific role of amygdala remains inconclusive^7,8^. Although exploratory in nature, our connectivity findings suggest that alterations in pathways involving mPFC and adjacent ACC, rather than amygdala, might be related to anxiety symptoms in IBS.

This study is not without limitations. We based our subgrouping of patients with high and low anxiety symptom severity on HADS scores. While we applied a conservative cut-off, HADS does not allow a proper psychiatric diagnosis. Future research addressing the association between brain alterations, including biochemical changes, and psychiatric comorbidity in disorders of brain-gut communication should implement diagnostic tools such as semi-structured interviews and clinical judgment^36^. Further, the qMRS protocol applied is optimized for the detection of GABA+. Although the signal-to-noise benefits achieved by a large voxel appeared to provide adequate sensitivity for Glx, we cannot rule out that our findings were affected by the chosen methodological approach. In addition, mPFC and ACC have previously been proposed to possess shared, but also distinct functional properties^9^. This is important not only with respect to spectroscopic findings, but also to connectivity analyses using a custom-built mPFC ROI, which corresponded to the voxel applied in qMRS, yet partly overlapped with the rostral proportion of ACC. Future research is needed, using a smaller voxel size for spectroscopic measures to allow targeting these prefrontal subregions specifically. In the current analysis, data were not adjusted for possibly divergent contributions of grey matter, white matter and cerebrospinal fluid to the voxel under investigation in patients and controls. Based on our previous experience applying quantitative MRI (qMRI) for tissue classification^37^, we decided not to include qMRI in the current protocol. Of note, cortical GABA concentrations are reportedly higher in grey relative to white matter and concentrations are negligible in cerebrospinal fluid^38,39^. Evidence from structural brain imaging in IBS supports decreased prefrontal grey matter density in patients^40,41^, which would rather suggest a relatively lowered concentration of the inhibitory neurotransmitter in the voxel of interest. While we cannot fully exclude that group differences observed in GABA+ concentrations may have been affected by distinct features in tissue contribution, these previous findings support that increased prefrontal GABA+ in IBS as observed herein, is unlikely to be mainly attributable to differences in tissue contribution between women with IBS and healthy women. Our study protocol did not include measurements of a control region such as the occipital cortex to substantiate the specificity of effects to the PFC observed herein. However, recently published data from our own group addressing insular metabolite concentrations in IBS patients lend support for such specificity^16^. Precisely, the observation of lowered Glx, yet unaltered GABA+ concentrations, in bilateral anterior insulae in patients suggests region-, as well as symptom-specific alterations rather than a general and widespread neurotransmitter dysbalance in IBS in support of GABAergic abundance specific to PFC in patients with high anxiety severity. Regarding the sample under investigation, our findings were derived from women only, limiting generalizability to men with IBS. Given evidence of sex-related differences in brain function in patients with IBS^42^, more research including both women and men is needed, which would also allow the investigation of putative sex-/gender-specific alterations of prefrontal excitatory and inhibitory neurotransmission. Finally, a small proportion of patients were taking antidepressant medication. However, our additional analyses excluding these patients confirmed our findings of increased GABA+ levels in IBS patients with more severe anxiety symptoms. Therefore, it appears unlikely that the observed alterations were primarily attributable to centrally-acting pharmacological agents.

### Conclusions and future directions

Our findings provide first evidence that dysfunctional prefrontal GABAergic neurotransmission and aberrant mPFC – ACC connectivity may form independent biochemical and functional substrates of increased anxiety as a frequent psychiatric comorbidity in IBS. Future multimodal studies including adequate disease control groups, especially patients with anxiety disorders but without gastrointestinal symptoms, could provide further insights into the specificity of and relation between altered prefrontal inhibitory neurotransmission, corticolimbic connectivity, psychological symptoms, and dysfunctional brain-gut communication. This is of particular importance given bidirectional brain-gut interactions to appear of crucial relevance beyond gastrointestinal complaints. Accumulating evidence, primarily from preclinical models, supports a key role of the gut microbiome and its communication pathways in shaping neural mechanisms, including brain biochemistry^43,44^, and behavior^45^. Findings suggest a putative role of microbiome-gut-brain pathways also in neuropsychiatric diseases, including anxiety and depression^46^. A recent experimental human model supports a close association between the microbial composition, brain structure and neural processing of emotional stimuli in healthy individuals^47^, extending evidence derived from elegant translational models^48^ or correlational approaches^49^. Together with these promising findings, our observations warrant further multimodal research approaches to help shed light on the complex interplay of central and peripheral mechanisms at the interface of neurogastroenterology and psychiatry.

## Methods

### Participants

Based on the high female preponderance in IBS^50^ and increased symptoms of anxiety and depression in women^51^, only female participants were included in this study. A total of 73 right-handed women (mean age, 31.73 ± 8.86 years) with a diagnosis of IBS based on Rome III diagnostic criteria were referred to the gastrointestinal unit of the Linköping University Hospital, Sweden, and 38 right-handed female healthy controls (HCs; mean age 33.45 ± 10.99 years) were recruited by local advertisement. Patients underwent a standard clinical examination to exclude organic gastrointestinal diseases and a laboratory examination was performed. Celiac disease was excluded on the basis of transglutaminase antibodies, and inflammatory bowel disease was excluded by f-calprotectin test. Exclusion criteria were metabolic, neurological, or severe psychiatric disorders (e.g., schizophrenia), smoking, claustrophobia, pacemaker, large tattoos, and metal implants. Of note, patients were not encouraged to refrain from antidepressant medication when enrolled in the study. In HCs, a medical history of GI disturbances, psychiatric conditions, or centrally-acting medication were exclusionary. Informed written consent was obtained from all participants and the study was approved by the Regional Research Committee for Ethical Issues at the Faculty of Health Sciences, Linköping, Sweden (Number: 2013/111-31) and conducted in accordance with the Declaration of Helsinki.

### Questionnaires

Prior to the study day, all participants completed the following questionnaires for the assessment of anxiety and depression symptom severity and of disease-related measures in their home environment. This approach was chosen to exclude possibly confounding effects induced by the scanner setting, such as an increase in distress^52^ with a putative impact on measures of anxiety and depression symptom severity.

### Hospital Anxiety and Depression Scale (HADS)

The Hospital Anxiety and Depression Scale (HADS) was used to assess symptoms of anxiety and depression^53^. The self-assessment questionnaire consists of 7 items per subscale scored on a 4-point scale, with each sum score ranging from 0 to 21. Cut-off scores are defined as ≥8 for suspicious and ≥11 for definite cases of anxiety and depression, respectively^34,53^.

### Visceral Sensitivity Index (VSI)

The Visceral Sensitivity Index (VSI) was implemented to measure GI symptom-specific anxiety^54^. This 15-item self-report questionnaire assesses cognitive, emotional, and behavioural responses to fear of GI symptoms. Sum scores range from 0 to 75 with higher scores indicating more severe symptom-specific anxiety.

### IBS Severity Scoring System (IBS-SSS)

The IBS Severity Scoring System (IBS-SSS) was used to evaluate the severity of abdominal pain, distension, stool frequency and consistency, and interference with life^55^. Sum scores ranging from 0 to 500 indicate mild (75–175), moderate (175–300), or severe (>300) IBS.

### Brief Pain Inventory (BPI)

The Brief Pain Inventory (BPI) was implemented to assess pain intensity and interference with functional and emotional domains^56^. Sum scores range from 0 to 40 and 0 to 70 for pain intensity and interference, respectively, with higher scores indicating higher levels of intensity and interference.

### MR data acquisition and analyses

Participants were instructed to refrain from consuming alcohol and using sleep or pain medication for at least 24 hours and to fast for at least four hours before MR data acquisition. All MR measurements were conducted using a 32-channel head coil on a 3 T Philips Ingenia MR-system (Philips Healthcare, Best, Netherlands). An initial structural brain scan was acquired to exclude brain abnormalities and allow accurate voxel placement for subsequent spectroscopy measures based on individual T_1_-weighted images.

**Figure 5:**
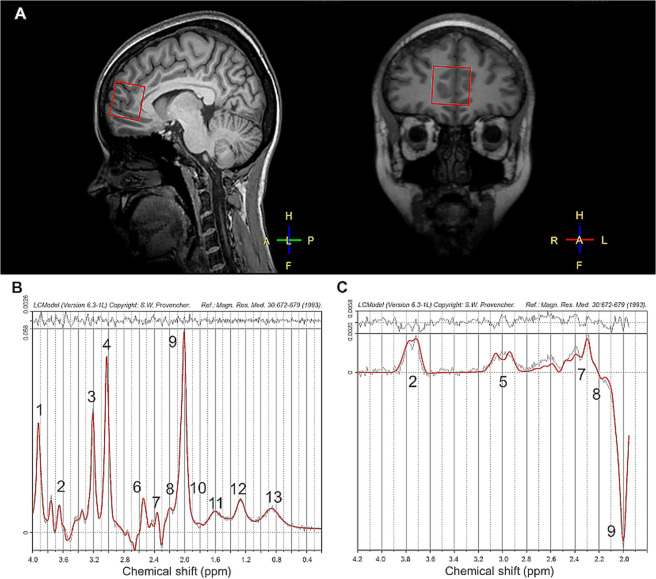
Typical qMRS volume of interest (voxel size 3 × 3 × 3 cm^3^) placement in the mPFC (**A**), representative spectra with LCModel fitting, depicting an averaged MEGA-PRESS OFF spectrum for Glx extraction (labeled as 2 and 7, extracted solely from OFF-spectra (**B**)) and a difference spectrum for the extraction of GABA+ (**C**) from a healthy volunteer (gray line: post-processed spectra prior to fitting; red line: LCModel fit). Residuals are shown at the top of each panel. Assignments: 1, Creatine (-^2^CH_2_-); 2, Glx (-^2^CH-); 3, Choline (-N(CH_3_)_3_); 4, Creatine (-N(CH_3_)); 5, GABA+ (-^4^CH_2_-); 6, tNA (-^3^CH_2_-); 7, Glx (-^4^CH_2_-); 8, GABA+ (-^2^CH_2_-); 9, tNA (-^2^CH_3_); 10. GABA+ (-^3^CH_2_-); 11–13, Macromolecules and lipids, -CH_2_-)^62^. Abbreviations: Glx, glutamate + glutamine; GABA+, γ-Aminobutyric acid (+macromolecule signal); tNA, total N-acetylaspartate (NAA + NAAG).

Following structural MRI, qMRS data were acquired using a MEGA-PRESS pulse sequence^57,58^ with the following parameters: TR/TE = 2000/68 ms, edited pulses ON at 1.90ppm and OFF at 7.46ppm, water suppression MOIST, 40 dynamics with a voxel of 30 × 30 × 30 mm^3^ placed in the mPFC (Fig. 5A). Subsequently, a 2-dynamic unsuppressed water reference measurement was collected to obtain a reference of water in the tissue within the voxel used, for the purpose of water scaling and phasing. Data were individually (*i*.*e*., data from each time point) phase-corrected^59^ and frequency-aligned based on the water residual in the water-suppressed data. A difference spectrum was computed by subtracting the average OFF-spectrum (Fig. 5B) from the average ON-spectrum and used as input to LCModel^60^ (Version 6.3-1L) to compute GABA+ concentrations^61^ (Fig. 5C). Analysis of OFF-spectrum dynamics was used to assess concentrations of Glx (Glu + Gln)^62^. The combined measure Glx rather than Glu and Gln separately was assessed based on the similar molecular structure of these metabolites, resulting in spectra with substantial overlap due to scalar couplings. At the field strength applied and with a protocol optimized for the detection of GABA+, a reliable distinction of Glu and Gln spectra is challenging^63^, limiting the interpretation of findings on these metabolites separately. Therefore, concentrations of Glx representing the entire pool of Glu and Gln in the voxel under investigation are reported.

In addition to the primary outcome parameters GABA+ and Glx, concentrations of total N-acetylaspartate (tNAA), Creatine (tCr) and Choline (Cho) as putative markers of neuronal health^64^ and neuro-inflammation^65,66^ were extracted from OFF spectra for exploratory between-group comparisons and correlational analyses. Data were analysed with LCModel (v.6.3-1L) using the most current basis sets from the laboratory of Dydak *et al*.^67^ with the following parameters: SPTYPE = mega-press-3, PPMST = 4.2, PPMEND = 1.95, DOWS = T, otherwise default values. All concentrations were water-scaled using the water reference, resulting in concentrations with absolute units of mM. Concentrations were not adjusted for contributions of grey matter, white matter and cerebrospinal fluid in the voxel of interest.

Spectra were quality controlled based on linewidth and data with a full-width half-maximun (FWHM) above 0.1 ppm were discarded (5 IBS, 4 HCs). In addition, each spectrum was visually inspected and datasets were excluded when lipid contamination obscuring other signals in the spectra was detected (4 IBS, 2 HCs), resulting in a final sample of N = 64 IBS patients (mean age, 31.55 ± 8.77 years) and N = 32 HCs (mean age 34.16 ± 10.72 years) for statistical analyses. For all included datasets, estimated relative standard deviations (Cramér-Rao lower bounds, CRLB) were <10% SD for GABA+ and for Glx, respectively.

Subsequent to spectroscopic measures, ten-minute eyes-closed resting-state fMRI data were acquired using a single-shot gradient-echo echo-planar imaging sequence with the following parameters: TR/TE = 2000/37 ms, voxel size 3.59 × 3.59 × 4.00 mm^3^, 28 slices and SENSE factor 2.00, which effectively covered the entire brain. Data were reconstructed on the scanner and preprocessed using SPM8 (Wellcome Trust Centre for Neuroimaging, UCL, London, UK) implemented in MATLAB R2015b (MathWorks, Natick, MA, USA). Images were realigned using the INRIalign toolbox^68,69^, and translation and rotation parameters were examined to exclude head motion exceeding one voxel in any direction. Functional images were spatially normalized into standardized Montreal Neurological Institute (MNI) space and smoothed with an 8 mm FWHM Gaussian kernel.

FC analysis was carried out in the CONN functional connectivity v17b toolbox^70^; (http://www.nitrc.org/projects/conn) on 95 of the 96 subjects, as one IBS patient was missing fMRI data. Data were submitted to the CONN standard denoising step, including band-pass filtering (0.008–0.09 Hz) and linear detrending. Motion regressors were included for all subjects. For first-level statistical analysis, a bivariate correlation using a weighted general linear model and hemodynamic response function weighting was conducted. Three separate ROI-to-ROI (region of interest) analyses were performed from a custom-built mPFC cubic voxel, designed to correspond to the qMRS voxel regarding size and approximate location, to bilateral amygdala, insula, and ACC, respectively. ROIs for amygdala, insula and ACC were selected from the Harvard-Oxford parcellated grey-matter atlas^71–74^. For these exploratory ROI-to-ROI analyses, significance was taken at *p* < 0.05 using FDR correction. Post-hoc results were considered significant at *p* < 0.05.

### Statistical analyses

Statistical analyses were performed using the IBM SPSS Statistics 25 software (IBM Corporation, Armonk, NY, USA). Shapiro-Wilk test revealed non-normal distribution of questionnaire data in HCs. Therefore, non-parametric tests were applied with Mann-Whitney *U*-test to assess differences between IBS patients and HCs and Spearman’s Rho for correlational analyses. To further elucidate the relation between neurotransmitter concentrations and psychological symptom severity in IBS, patient subgroups were defined based on HADS scores ≥11 as a well-established cut-off for the detection of anxiety or depression caseness^34,53^. Between-group analyses were performed using Kruskal-Wallis test followed by post-hoc *U*-tests with Bonferroni correction. Mean FC values from CONN ROI-to-ROI analyses were extracted and entered into exploratory group, subgroup, and correlational analyses. Finally, multiple regression analyses with neurotransmitter and FC data entered as predictor variables were conducted in patients to further explore the link between neurotransmission, resting state connectivity and psychological symptom severity. Alpha-levels for statistical tests were set at *p* < 0.05. Exact two-tailed *p* values are provided, using the Monte Carlo method for group comparisons, and applying bootstrapping for correlational analyses, both with 10.000 iterations and a confidence level of 99%. Results from group comparisons are reported as median and interquartile range (IQR).

The datasets generated and analysed during the current study are available from the corresponding author on reasonable request.

## Supplementary information

Elucidating the putative link between prefrontal neurotransmission, functional connectivity, and affective symptoms in irritable bowel syndrome: Supplementary information
