# Supplementary material for: Elucidating the putative link between prefrontal neurotransmission, functional connectivity, and affective symptoms in irritable bowel syndrome
Source: Sci Rep. 2019 Sep 19;9:13590. doi: 10.1038/s41598-019-50024-3 (PMC6753205; doi:10.1038/s41598-019-50024-3)
Supplement: Supplementary file 1 — Elucidating the putative link between prefrontal neurotransmission, functional connectivity, and affective symptoms in irritable bowel syndrome: Supplementary information [file 41598_2019_50024_MOESM1_ESM.pdf]

Elucidating the putative link between prefrontal neurotransmission, functional connectivity, and affective symptoms in irritable bowel syndrome

Adriane Icenhour*[a,b,f], Sofie Tapper[b,c], Olga Bednarska[a], Suzanne T Witt[b], Anders Tisell[b,d,e], Peter Lundberg[b,d,e], Sigrid Elsenbruch[f], Susanna Walter[a,b]

[a] Department of Gastroenterology and Department of Clinical and Experimental Medicine, Linköping University, Linköping, Sweden
[b]Center for Medical Image Science and Visualization (CMIV), Linköping University, Linköping, Sweden
[c] Department of Medical and Health Sciences, Linköping University, Linköping, Sweden
[d]Department of Radiation Physics, Department of Medical and Health Sciences, Linköping University, Linköping, Sweden
[e]Department of Radiology, Department of Medical and Health Sciences, Linköping University, Linköping, Sweden
[f]Institute of Medical Psychology and Behavioral Immunobiology, University Hospital Essen, University of Duisburg-Essen, Essen, Germany

Supplementary information

Supplementary table S1: Sample characterization and group comparisons of clinical characteristics and psychological measures

Supplementary information S2: (a) Group and (b) subgroup comparisons of neurotransmitter levels excluding patients on antidepressant treatment

Supplementary table S3: Group comparisons of mPFC N-acetylaspartate, Creatine and Choline concentrations.

Supplementary table S4: Correlations between neurotransmitter concentrations, clinical characteristics, and psychological measures.

Supplementary table S1

Sample characterization and group comparisons of clinical characteristics and psychological measures.

	IBS (N = 64)	HCs (N = 32)	*p*
**Age (years)**	30.00 (24.25 – 36.75)	32.5 (24.00 – 45.25)	0.330
**Intestinal symptoms (IBS-SSS)**	329.00 (275.00 – 384.00)	4.00 (0.00 – 23.00)	< 0.001
**Pain intensity (BPI)**	15.00 (11.00 – 21.50)	0.00 (0.00 – 0.00)	< 0.001
**Pain interference (BPI)**	32.00 (11.00 – 45.50)	0.00 (0.00 – 0.00)	< 0.001
**Symptom-specific anxiety (VSI)**	44.50 (32.00 – 57.00)	1.00 (0.00 – 3.00)	< 0.001
**Anxiety (HADS)**	10.00 (8.00 – 14.00)	3.50 (2.00 – 5.00)	< 0.001
**Depression (HADS)**	5.00 (3.00 – 8.75)	1.00 (0.00 – 2.00)	< 0.001

Data are given as median and interquartile ranges are provided. Abbreviations: BPI, brief pain inventory; HADS, Hospital anxiety and depression scale; HCs, Healthy Controls; IBS, Irritable Bowel Syndrome; IBS-SSS, IBS symptom severity score intestinal; VSI, visceral sensitivity index.

Supplementary information S2

a) **Group comparisons of neurotransmitter levels excluding patients on antidepressant treatment**

To exclude possible confounding effects of antidepressant medication on mPFC neurotransmission, additional analyses were performed in which 21 patients who had reported SSRI or low-dose TCA medication intake at the time of participation were excluded. Mann-Whitney *U*-tests comparing only patients free from antidepressant medication (N = 43) with HCs (N = 32) confirmed no group differences in either GABA+ or Glx concentrations (Supplementary table S2).

Table S2: Comparison of mPFC GABA+ and Glx concentrations in patients free from antidepressant treatment and HCs.

	**IBS (N = 43)**	**HCs (N = 32)**	***p***
**mPFC GABA+**	0.71 (0.66 – 0.76)	0.71 (0.62 – 0.75)	0.509
**mPFC Glx**	4.71 (4.01 – 5.12)	4.44 (3.81 – 5.23)	0.495

Data are given as median and interquartile ranges are provided. Abbreviations: mPFC, medial prefrontal cortex; GABA+, γ-aminobutyric acid+ macromolecule signal; Glx, glutamate+glutamine.

b) Subgroup comparisons of GABA+ levels excluding patients on antidepressant treatment

As performed in the full sample, women with IBS and free of antidepressant treatment were sub-grouped based on HADS anxiety scores, resulting in groups of 18 IBS^+ patients (*i.e.*, 11 patients in this group were excluded due to medication intake) and 25 IBS^- patients (*i.e.*, 9 patients were excluded). These data were subjected to a Kruskal-Wallis test followed by post hoc *U*-tests. Kruskal-Wallis-test revealed a trend-level group effect ($X^2 = 4.802$; $p < 0.10$).

Exploratory post hoc *U*-tests supported enhanced GABA+ levels in IBS+ compared to IBS- patients ($U = 140.00$; $p = 0.039$) and a trend towards higher GABA+ levels in IBS with high anxiety symptom severity compared to HCs ($U = 200.00$; $p = 0.077$), essentially confirming the results in the full sample while failing to reach statistical significance after correction for multiple comparisons.

Supplementary table S3

Group comparisons of mPFC N-acetylaspartate, Creatine and Choline concentrations.

	IBS (N = 64)	HCs (N = 32)	*p*
**N-acetylaspartate**	4.95 (4.53 – 5.89)	4.75 (4.18 – 5.44)	0.098
**Creatine**	5.00 (4.41 – 5.58)	4.51 (4.00 – 5.46)	0.105
**Choline**	1.04 (0.94 – 1.19)	0.99 (0.87 – 1.23)	0.408

Data are given as median and interquartile ranges are provided. Abbreviations: HCs, Healthy Controls; IBS, Irritable Bowel Syndrome.

Supplementary table S4

Correlations between neurotransmitter concentrations, clinical characteristics, and psychological measures.

	Age	GABA+	Glx	Anxiety	Depression	GI-specific anxiety	Symptom severity	Pain intensity	Pain interference
**Age**	1	-0.089	-0.044	-0.144	-0.009	-0.184	-0.150	-0.018	-0.092
	96	0.425	0.698	0.198	0.937	0.099	0.180	0.874	0.412
**GABA+**		1	0.094	**0.280****	**0.222***	0.149	0.144	0.083	0.066
		96	0.399	**< 0.01**	**< 0.05**	0.181	0.198	0.460	0.559
**Glx**			1	0.070	-0.038	0.066	0.152	0.097	0.098
			96	0.534	0.732	0.553	0.174	0.386	0.379
**Anxiety**				1	**0.805*****	**0.735*****	**0.715*****	**0.724*****	**0.735*****
				96	**< 0.001**	**< 0.001**	**< 0.001**	**< 0.001**	**< 0.001**
**Depression**					1	**0.659*****	**0.650*****	**0.686*****	**0.721*****
					96	**< 0.001**	**< 0.001**	**< 0.001**	**< 0.001**
**GI-specific anxiety**						1	**0.838*****	**0.769*****	**0.752*****
						96	**< 0.001**	**< 0.001**	**< 0.001**
**Symptom severity**							1	**0.809*****	**0.839*****
							96	**< 0.001**	**< 0.001**
**Pain intensity**								1	**0.881*****
								96	**< 0.001**
**Pain interference**									1
									96

Spearman’s rank correlations between Glx and GABA+ concentrations, psychological and disease-related measures conducted in the full sample. Abbreviations: GABA+, γ-aminobutyric acid+ macromolecule signal; GI, gastrointestinal; Glx, glutamate+glutamine.
